# Transcriptome Analysis of Protocatechualdehyde against *Listeria monocytogenes* and Its Effect on Chicken Quality Characteristics

**DOI:** 10.3390/foods12132625

**Published:** 2023-07-06

**Authors:** Sichen Liao, Lu Tian, Qi Qi, Lemei Hu, Minmin Wang, Chang Gao, Haoyue Cui, Zhongchao Gai, Guoli Gong

**Affiliations:** School of Food Science and Engineering, Shaanxi University of Science and Technology, Xi’an 710021, China; 13772639155@163.com (S.L.); 15129053823@163.com (Q.Q.); 13992194188@163.com (L.H.); 15891599270@163.com (M.W.); gaochang5820@163.com (C.G.); 18791749131@163.com (H.C.); zhongchaogai@sust.edu.cn (Z.G.); guoligong@sust.edu.cn (G.G.)

**Keywords:** protocatechualdehyde, *Listeria monocytogenes*, antibacterial activity, action mechanism, transcriptome, cooked chicken breast meat quality

## Abstract

The development of natural antimicrobial agents offers new strategies for food preservation due to the health hazards associated with the spoilage of meat products caused by microbial contamination. In this paper, the inhibitory mechanism of protocatechualdehyde (PCA) on *Listeria monocytogenes* was described, and its effect on the preservation of cooked chicken breast was evaluated. The results showed that the minimal inhibitory concentration (MIC) of PCA on *L. monocytogenes* was 0.625 mg/mL. Secondly, PCA destroyed the integrity of the *L. monocytogenes* cell membrane, which was manifested as a decrease in membrane hyperpolarization, intracellular ATP level, and intracellular pH value. Field emission gun scanning electron microscopy (FEG-SEM) observed a cell membrane rupture. Transcriptome analysis showed that PCA may inhibit cell growth by affecting amino acid, nucleotide metabolism, energy metabolism, and the cell membrane of *L. monocytogenes*. Additionally, it was discovered that PCA enhanced the color and texture of cooked chicken breast meat while decreasing the level of thiobarbituric acid active substance (TBARS). In conclusion, PCA as a natural antibacterial agent has a certain reference value in extending the shelf life of cooked chicken breast.

## 1. Introduction

Meat has been found as the main source of human protein, whereas fresh meat is easily impacted by a range of elements (e.g., microorganisms, light, and heat) and deteriorates at ambient temperature. Spoiled meat products are not edible, and the spoilage of meat products will pose a safety hazard of food poisoning, thus leading to waste of resources and environmental pollution. The World Health Organization (WHO) estimates that more than 150 million cases of diarrheal diseases occur worldwide each year, and the above cases are correlated with food contaminated by a variety of pathogenic microorganisms. Common food-borne pathogens in meat consist of *Escherichia coli*, *Salmonella*, *Listeria monocytogenes*, *Staphylococcus aureus*, *Pseudomonas adaceae*, etc.

*L. monocytogenes* refers to a facultative anaerobic gram-positive bacillus [1]. *L. monocytogenes*, a food-borne pathogen with strong vitality, can survive at a temperature of 0–45 °C. It has been termed the refrigerator killer. It is extensively found in nature, primarily in livestock and poultry, easy to pollute meat and meat products, dairy products, eggs, vegetables, fruits and vegetables, aquatic products, and other foods, as well as capable of spreading through unclean food [2]. Humans infected with *L. monocytogenes* can cause coma, dyspnea, sepsis, meningitis, miscarriage, and even death in severe cases [3]. Over the past few years, food poisoning incidents due to the *L. monocytogenes* contamination of meat products have been reported frequently. Between 2017 and 2018, there was an outbreak of listeriosis involving meat products in South Africa, with nearly 1060 cases of infection and 216 deaths. It has been recognized as the largest listeriosis outbreak in recorded history [4]. The European Food Safety Authority report suggested that over 200 cases of listeriosis were recorded in Spain in 2019, which were related to the cold roast pork of the “La Mecha” brand; the deaths of three elderly people, and the loss of six pregnant women with babies were reported [5].

Currently, heat has been commonly used to kill *L. monocytogenes.* However, *L. monocytogenes* can also survive in general heat processing treatments. Even if the product has been inactivated through adequate thermal processing, the product may be contaminated again. Accordingly, it is extremely important to prevent secondary contamination after steaming. To delay the deterioration of meat products and extend their shelf life, a wide variety of food additives have been often used to inhibit food-borne pathogenic bacteria to a certain extent. Nitrates have been found as one of the vital food additives, whereas the presence of carcinogenic compounds in nitrites is labeled as unhealthy [6]. Impacted by the potentially toxic side effects and residues of chemical preservatives and due to the problem of environmental pollution, the search for bioactive substances from natural resources to replace chemical substances as natural preservatives have aroused rising attention [7]. It is generally known that natural plant extracts with green, safe, efficient, good antioxidant effect and broad-spectrum antibacterial effect have become a hot spot for research and are gradually applied in various food fields. Over the past few years, Protocatechualdehyde (PCA), a natural phenolic acid compound, has aroused wide attention for its multiple effects. There is some scientific value in using PCA as a natural food additive to increase product shelf life, and it has potential applications in food preservation.

PCA is a water-soluble phenolic acid compound which has been detected in a variety of Chinese herbs (e.g., gall leaves, salvia roots, purple flower holly leaf) [8]. According to preliminary studies, PCA has various biological activities such as antibacterial, antioxidant [9], anti-tumor [10], and cardiovascular protection [11], and its antibacterial effect has become the focus of research. For example, PCA has a certain inhibitory effect on the growth of eight pathogens, including *Yersinia enterocolitica*, *Cronobactersakazakii*, *S. aureus*, and *Bacillus cereus* [12,13,14,15]. PCA has been employed as a dietary supplement in several health foods. However, the effectiveness of PCA on food-borne pathogenic bacteria and its application in meat preservation have been rarely reported.

Transcriptomics is a comprehensive approach to analyzing the entire set of transcripts of specific cells, tissues, or whole organisms under certain specific physiological conditions, which can resolve the changes in gene expression of microorganisms at the molecular level and reveal the molecular mechanisms involved in biological processes [16]. Therefore, transcriptomics has been widely used in recent years to study the mechanism of action of natural plant-derived extracts against food-borne pathogenic bacteria [17]. For example, Li et al. found that linalool disrupted the extracellular lipopolysaccharide synthesis pathway of *Pseudomonas fragilis* by RNA-seq techniques and activated fatty acid metabolism and ribosome function to compensate for cell membrane damage, while linalool severely interfered with energy metabolism, causing overexpression of various intracellular ATP synthases and ATP transporter enzymes, leading to ATP overconsumption [18]. Therefore, this study used in vitro bacterial inhibition assays to evaluate the antimicrobial mechanism of the natural antimicrobial substance PCA against *L. monocytogenes* and to reveal its molecular mechanism at the mRNA level based on transcriptomics techniques. Finally, PCA was applied as an antimicrobial agent to act on cooked chicken breast meat to detect physiological and biochemical parameters, analyze its preservative effect and theoretically support the development of effective natural preservative preservatives for meat products. This study aims to investigate the antibacterial effect of natural plant extracts on food-borne pathogenic bacteria and to lay a theoretical foundation for the application of food antimicrobial agents.

## 2. Materials and Methods

### 2.1. Reagents

PCA [HPLC ≥ 98%, CAS: 139-85-5] was provided by Bioengineering Co., Ltd. (Shanghai, China). PCA stock solution was prepared with ethanol as the solvent and then dissolved in the following solutions, including Mueller-Hinton II Broth (CA-MHII), Brain-Heart Infusion Broth (BHI), and Phosphate adjusted Buffered Saline (PBS), with the ethanol concentration of 2% in the final reaction system. All the other chemicals adopted to prepare different buffers (including Na_2_HPO_4_⋅12H_2_O, KH_2_PO_4_, K_2_HPO_4_, NaCl, KCl, and MgCl_2_) were of analytical grade.

### 2.2. Bacterial Strain and Culture Conditions

*L. monocytogenes* ATCC 19114 was purchased from the American Type Culture Collection (ATCC, Beijing, China) and stored in a glycerine bottle at −80 °C. These cells were inoculated in BHI broth for activation and then incubated at 37 °C for 8 h to allow bacterial growth to the logarithmic growth phase. The final bacterial cultures of *L. monocytogenes* were employed for all tests in the study. Ampicillin (final concentration of 0.1 mg/mL) was the reference antibiotic.

### 2.3. Cooked Chicken Breast Pretreat Ment

The fresh chicken breast meat was ground twice in a muscle grinder fitted with 6 mm and 3.2 mm well plates, respectively, and the ground meat was well mixed. Subsequently, the meat was cooked in boiling water and then sterilized under a UV lamp for 30 min. The cell pellets were centrifuged (8000× *g*, 5 min), and PCA solution containing 0, minimum inhibitory concentration (MIC) and 2 × MIC with bacterial cells was prepared. A total of 1 mL of the solution was aspirated and spread evenly on the surface of 100 g of cooked chicken breast meat. Next, the meat was wrapped with a PVC film and placed in a refrigerator at 4 °C. Finally, the preservation effect of PCA on cooked chicken breasts was evaluated by detecting the total number of colonies and mass changes at 1, 3, 5, and 7 days.

### 2.4. Minimum Inhibitory Concentration Determinations

The broth microdilution method of Wiegand et al. was used with some modifications to determine the MIC of PCA on the growth of *L. monocytogenes* [19]. PCA solution with a concentration of 5 mg/mL was prepared using a CA-MHII medium, and then a final PCA concentration of 5 to 0 mg/mL was obtained according to the two-fold dilution method. Next, 100 μL of bacterial culture and 100 μL of PCA solution at different concentrations were added dropwise to a 96-well plate (Nunc, Copenhagen, Denmark). Afterward, the plate was incubated at 37 °C for 8 h, and the absorbance values were examined at 600 nm.

### 2.5. Growth Curves

The growth curves were generated using the method of Tian et al. with some modifications [12]. The PCA solutions were prepared with different concentrations of BHI containing 2% ethanol to a final concentration of 2 × MIC to 1/64 × MIC. Subsequently, the OD_600_ values were measured every 1 h in the incubator at 37 °C using a full wavelength scanning multifunctional reader (Varioskan Flash, Thermo Fisher, Vantaa, Finland) to monitor the cell growth for 24 h.

### 2.6. Membrane Integrity and Membrane Potential

The cell suspensions were treated with 0, MIC, and 2 × MIC of PCA solution (PBS containing 2% ethanol) and incubated for 2 h at 37 °C following the modified protocol of Guo et al. [20]. Afterward, 1 mM of the fluorescent probe bis-(1,3-dibutylbarbituric acid) trimethoprim [DiBAC_4_(3); Molecular Probes, Sigma, St. Louis, MO, USA] was added to the cell suspension and treated for 10 min under light-protected conditions. Fluorescence intensity at 492 nm excitation/515 nm emission wavelengths was measured using a multi-mode reader (Synergy H1, BioTek, Winooski, VT, USA).

### 2.7. Determination of Intracellular ATP

The intracellular ATP was detected using the ATP detection kit (Beyotime bioengineering institute, Shanghai, China) based on the method presented by Bajpai et al. [21]. PCA solutions of 0, MIC, and 2 × MIC were prepared with PBS solution supplemented with 2% ethanol, and bacterial cells were resuspended and incubated at 37 °C for 2 h. Chemiluminescence intensity was measured with a multi-mode reader (Synergy H1, BioTek, Winooski, VT, USA).

### 2.8. Intracellular pH_in_ Measurements

Fluorescently labeled cells were resuspended with 0, MIC, and 2 × MIC concentrations of PCA (supplemented with 2% ethanol) and incubated for 1 h protected from light. According to the method described by Tian et al. [12]. The fluorescence intensity of the samples was measured with the multifunctional enzyme marker (Synergy H1, BioTek, Winooski, VT, USA) under the excitation wavelengths set at 490 nm and 440 nm and under the emission wavelength at 520 nm, respectively. Furthermore, the standard test curve was generated, and the intracellular pH of the samples was determined.

### 2.9. Confocal Laser Scanning Microscopy (CLSM) Examinations

The LIVE/DEAD BacLight™ bacterial viability assay kit (Molecular Probes, Thermo Fisher, Waltham, MA, USA) was used to stain the bacteria according to the method described by Tian et al. [12]. PCA solutions (0, MIC, and 2 × MIC) were prepared with 0.85% NaCl solution containing 2% ethanol at various concentrations. Subsequently, bacterial cells were resuspended and incubated at 37 °C for 2 h. After the bacteria were resuspended with saline, 3 μL of SYTO/PI mix dye was added dropwise to each mL and then mixed for 10–15 min without being exposed to light. The bacteria were stained under a CLSM microscope (LSM800, Carl Zeiss, Yarra, Dulmen, Germany) to observe the cell membrane permeability.

### 2.10. Field Emission Gun Scanning Electron Microscopy (FEG-SEM) Analysis

The method presented by Su et al. was referenced and modified in this study [22]. Bacterial cells were treated with PCA solutions (supplemented with ethanol) at different concentrations of 0, MIC, and 2 × MIC for 4 h, washed three times with PBS solution and resuspended in 2.5% glutaraldehyde for 12 h. Subsequently, the bacteria were dehydrated with the ethanol solution with different concentration gradients for 10 min each time. Lastly, the suspensions were resuspended in isoamyl acetate for 30 min and then dried. The cell morphology was identified under a high-resolution field emission scanning electron microscopy (MLA 650, FEI, Hillsboro, OR, USA).

### 2.11. RNA Sequence and Bioinformatics Analysis

The bacterial suspensions were treated with different concentrations of 0 and MIC of PCA solution (PBS with 2% ethanol) for 2 h. The *L. monocytogenes* bacterial precipitate was then collected by centrifugation at 8000× *g* for 5 min at 4 °C, and the bacteria were washed 2–3 times with PBS. The Mackie Bio RNA extraction kit was used to extract RNA from *L. monocytogenes*. And sample quality control was then performed using Thermo NanoDrop One (Thermo Fisher Scientific, Waltham, MA, USA) and Agilent 4200 Tape Station (Agilent Technologies, Santa Clara, CA, USA). All RNA samples obtained had an A260/A280 ratio between 2.1 and 2.2, and the samples were of good quality. Afterward, ribosomal RNA was removed from the samples using the Ribo-Zero rRNA removal kit (Epicentre, San Antonio, CA, USA), and cDNA libraries were constructed from the purified RNA according to the NEBNext^®^ UltraTM II RNA Targeted Library Preparation Kit (Illumina, San Diego, CA, USA). The constructed libraries will be subjected to library quality control, and the libraries that pass the quality control will be sequenced by PE150 using Illumina’s high-throughput sequencing platform.

To ensure the quality of bioinformatics analysis, raw reads with low-quality were eliminated to obtain clean reads, and further analysis was predicated on these clean data.

### 2.12. Simulation Study of PCA on the Growth Inhibition of L. monocytogenes in the Cooked Chicken Breast Meat

The cooked chicken breast meat samples containing *L. monocytogenes* were diluted and then coated on BHI agar based on the method of Zhang et al. with some modifications [23]. The Box–Behnken test was performed to simulate the effect of PCA concentration (A), storage temperature (B), and storage time (C) on the total number of *L. monocytogenes* colonies. Moreover, tests were performed, and actual colony counts were recorded according to those listed in Table 1. Finally, the response surface methodology was used to simulate the growth of *L. monocytogenes* in the cooked chicken breast by PCA under different parameters.

### 2.13. Color Measurement

The color difference of the minced cooked chicken breast meat in the cold storage process was measured with a Cr-600 colorimeter (Minolta, Osaka, Japan) by the method of Cachaldora et al. [24]. A sample with a thickness of 1 cm was taken, and three parallels were set for the respective group of samples. Zero correction and whiteboard correction were conducted for the spectrocolorimeter, and the brightness value (L*), redness value (a*), and yellowness value (b*) of the respective group were examined on the 1, 3, 5, and 7 days.

### 2.14. Determination of Texture Profile Analysis (TPA)

Following the Novakovi et al. protocol with certain improvements, the cooked chicken breast was made into a cylinder of about 1 cm in height and 2 cm in diameter [25]. TPA was performed on cooked chicken breasts using a TA Plus physical property tester to determine the hardness, springiness, cohesiveness, chewiness, and resilience. The probe model was P/75, the compression ratio was obtained as 35%, the speed before and after the test was 1 mm/s, and the speed after the test was 5 mm/s.

### 2.15. Determination of Thiobarbituric Acid Reactive Substances (TBARS)

Referring to the method studied by Al-Hijazeen et al. [26], 2.0–2.5 g of cooked chicken breast meat samples were accurately weighed, 1.5 mL of Thiobarbituric acid (TBA) solution (1% TBA dissolved in 0.75 mol/L sodium hydroxide) and 8.5 mL Trichloroacetic acid (TCA) solution (2.5% TCA dissolved in 0.036 mol/L HCl) were added in turn. This was followed by mixing in a water bath at 100 °C for 30 min, and then immediately after cooling to room temperature in an ice water bath, 4 mL of supernatant was taken, 4 mL of chloroform was added, shaken thoroughly, and centrifuged at 3000× *g* for 5 min. Next, the supernatant was taken out to measure the absorbance ABS at a wavelength of 532 nm. The results were expressed as malondialdehyde (MDA) equivalents (mg/kg). The TBARS value was determined by TBARS (mg/kg) = *ABS/W* × 9.48.

### 2.16. Sensory Evaluation

Sensory evaluation of chicken breast patties treated with different concentrations of PCA was carried out according to our previous method to develop a 9-point happiness scale [27]. This was used to verify the effect of PCA on the color, texture, odor, taste, and overall appreciation of the chicken breast patties.

### 2.17. Statistical Analysis

Significance analyses were performed using SPSS software (version 19.0; SPSS Inc., Chicago, IL, USA). Data are expressed as mean ± standard deviation (*n* = 3), and differences between data that followed a normal distribution (Shapiro–Wilk’s test) were assessed using one-way ANOVA and Tukey’s tests. Differences between groups were verified using the Student’s *t*-test. Differences reached statistical significance when *p* ≤ 0.05.

## 3. Results and Discussion

### 3.1. MIC and Growth Curves of PCA to L. monocytogenes

The MIC of PCA against *L. monocytogenes* was obtained based on the broth microdilution method at 0.625 mg/mL. Tian et al. demonstrated the significant bacterial inhibitory effect of PCA on *Y. enterocolitica* BNCC 108930 by micro-broth dilution method with a MIC value of 0.3125 mg/mL [12]. Gutiérrez-Larraínzar et al. determined the inhibitory concentration of the microorganisms based on the standard microdilution method and obtained the MIC of PCA against *S. aureus*, *Bacillus cereus*, and *E. coli* as 0.9, 1.0 and 3.0 mg/mL, respectively [13]. Prachayasittikul et al. found that the MIC of PCA against *Plesiomonas shigelloides* was ≤60 µg/mL based on the agar dilution method [28]. In general, the results of this study using the broth microdilution method were consistent with the results achieved by others. The significant difference from the results of Prachayasittikul et al. might arise from the different methods applied and the variability of PCA on different strains, thus resulting in significant differences in the MIC.

**Figure 1:**
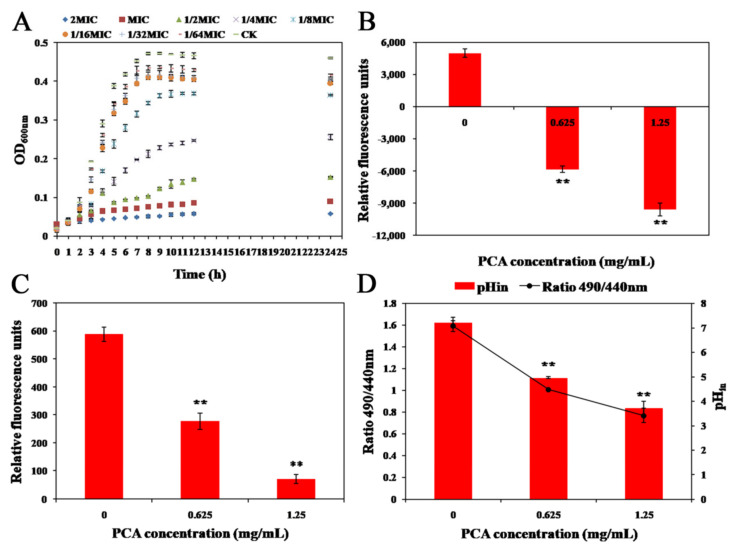
(**A**) The growth curves of *L. monocytogenes* ATCC 19114 exposed to PCA at different concentrations. (**B**) Effect of PCA on the membrane potential trend of *L. monocytogenes* ATCC 19114. (**C**) The effect of PCA on intracellular ATP concentration in *L. monocytogenes* ATCC 19114. (**D**) Effect of PCA on the pH_in_ of *L. monocytogenes* ATCC 19114 (** *p* < 0.05).

The growth curves of 1/64 × MIC to 2 × MIC were selected to assess the inhibitory effect of PCA on the growth of *L. monocytogenes*, and no regression analysis was performed for this part. As depicted in Figure 1A, the growth of *L. monocytogenes* became stable after 10 h with OD_600_ = 0.47. The growth of *L. monocytogenes* was affected by different concentrations of PCA. Compared with the negative control group, the bacterial optical density values were reduced by 2/3 and 1/2 in the 1/2 × MIC and 1/4 × MIC groups, respectively. But the bacterial optical density values in the MIC group were in a low-level linear trend for 24 h, indicating that PCA at MIC could completely inhibit its growth during the whole growth cycle. And overall, there was a significant dose-dependent relationship between the inhibitory activity of PCA against *L. monocytogenes*.

### 3.2. Membrane Potential

As part of the proton dynamic potential, the membrane potential not only characterizes the metabolic activity of bacterial cells but also plays a role in the synthesis of adenosine triphosphate (ATP). Changes in membrane potential are related to cell permeability, and changes in cell membrane potential can be captured by cell fluorescence intensity [29]. Figure 1B presents the changes in the membrane potential of *L. monocytogenes*. The cell-related fluorescence of DiBAC_4_(3) was significantly reduced by the PCA treatment (*p* < 0.05), and the intensity of fluorescence and hyperpolarization decreased with increasing PCA concentration. This result was achieved probably due to the effect of the PCA treatment on the structure and function of the *L. monocytogenes* cell membranes, increasing the potential difference between the inner and outer cell membranes. As a result, the cells were hyperpolarization, and the cell membrane integrity was impaired, thus leading to metabolic abnormalities [30].

### 3.3. Intracellular ATP Concentrations

The synthesis and catabolism of ATP are vital mechanisms for cells to maintain homeostasis and directly provide energy for cellular metabolism. It plays an essential role in cellular material transport, energy conversion, membrane potential regulation, and information transmission [31]. As depicted in Figure 1C, under normal conditions, the content of ATP in *L. monocytogenes* cells was relatively stable. After the PCA treatment was performed, the cell membrane of the bacterium was damaged, resulting in the cell membrane becoming unstable. As a result, the rate of intracellular ATP synthesis and ATP level would be reduced. Additionally, with the increase in the PCA concentration, the intracellular ATP content of *L. monocytogenes* decreased more significantly (*p* < 0.05). As revealed by the above results, PCA could affect the life activity of *L. monocytogenes* and promote or lead to cell death. Several studies also suggested that when cells are stimulated to apoptosis by the external environment, the accelerated rate of cell hydrolysis and respiration will be inhibited. To ensure the proton homeostasis of the cells, the intracellular ATP content will dramatically decrease [32].

### 3.4. Intracellular pH_in_ Measurements

pH_in_ plays an important regulatory role in cell growth, ion conversion, receptor-mediated signal transduction, enzyme activity, as well as cell adhesion. Intracellular pH changes were monitored by infiltrating esterase substrates with cFDA-SE [33]. Figure 1D presents the change of intracellular pH of *L. monocytogenes*, pH_in_ of normal cells was 6.82 ± 0.22 and decreased significantly to 3.18 ± 0.29 after the PCA treatment was performed (*p* < 0.05). The decrease in the intracellular pH of *L. monocytogenes* cells caused a decrease in the membrane proton pump function of the bacteria and irreversible denaturation of intracellular proteins and DNA. Thus, *L. monocytogenes* could not carry out normal metabolism and would eventually lead to death [33].

### 3.5. CLSM Observation

**Figure 2:**
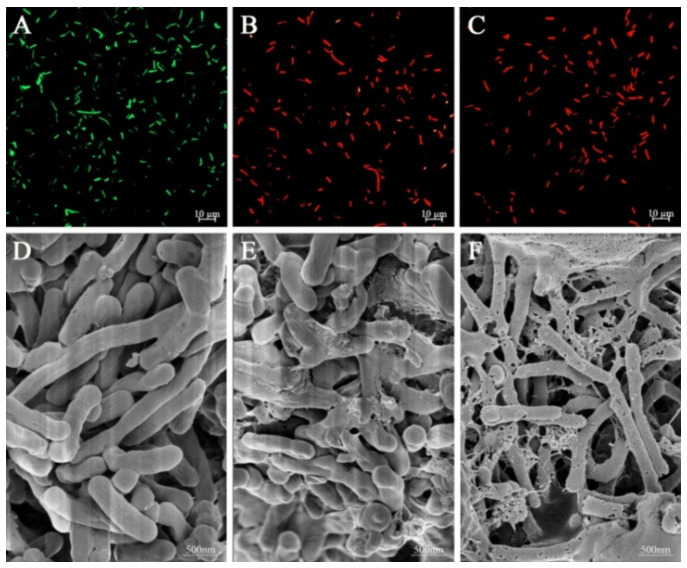
Effects of PCA on cell viability of *L. monocytogenes* ATCC 19114 by CLSM. *L. monocytogenes* cellstreated with (**A**) 2% ethanol, (**B**) MIC, and (**C**) 2 × MIC of PCA, respectively. The scanning electron micrographs of *L. monocytogenes* ATCC 19114 were based on different treatments. *L. monocytogenes* were treated with (**D**) 2% ethanol, (**E**) MIC, and (**F**) 2 × MIC of PCA, respectively.

Confocal laser fluorescence microscopy works based on two nucleic acid dyes, SYTO 9 and PI, which stain *L. monocytogenes* and fluoresce in different colors. SYTO 9 freely penetrates the cell membrane of all cells and fluoresces green, representing cell survival; PI, a small cationic molecule, only penetrates damaged cell membranes and fluoresces red, representing cell death [34]. Figure 2A–C present the results of fluorescence changes observed by CLSM. The untreated group of *L. monocytogenes* showed an intense green fluorescence. The field of view’s green fluorescence dramatically decreased as PCA concentration increased, but red fluorescence significantly increased. All *L. monocytogenes* exhibited red fluorescence in the field of view, as presented in Figure 2C. The above result suggested that PCA damaged the cell membrane permeability of *L. monocytogenes* while effectively inhibiting the growth and differentiation of cells; the degree of cell death increased with the increase in the PCA concentration. Furthermore, the leakage of content material and cellular deformation due to damaged cell membranes have been reported to induce cell death eventually [35].

### 3.6. FEG-SEM Observation

To more clearly observe the morphological alterations and cell membrane integrity of PCA-treated *L. monocytogenes* cells, FEG-SEM results were achieved, as presented in Figure 2D–F. Untreated *L. monocytogenes* exhibited an intact and smooth cell membrane morphology, thus showing a rounded rod shape. The surface of PCA-treated cells at MIC showed partial collapse, and the cytoskeleton was largely destroyed. And when the PCA concentration was increased to 2 × MIC, the *L. monocytogenes* cell membrane ruptured, and mucus-like material appeared in the interstitial space of the bacterium. The cell outline was severely deformed. This shows that the extent of *L. monocytogenes* cell membrane damage was more severe with the increase in PCA concentration. A similar effect was observed in PCA-treated *Y. enterocolitica* [12].

### 3.7. Transcriptome Analysis

#### 3.7.1. Global Analysis of Transcriptome Data

**Figure 3:**
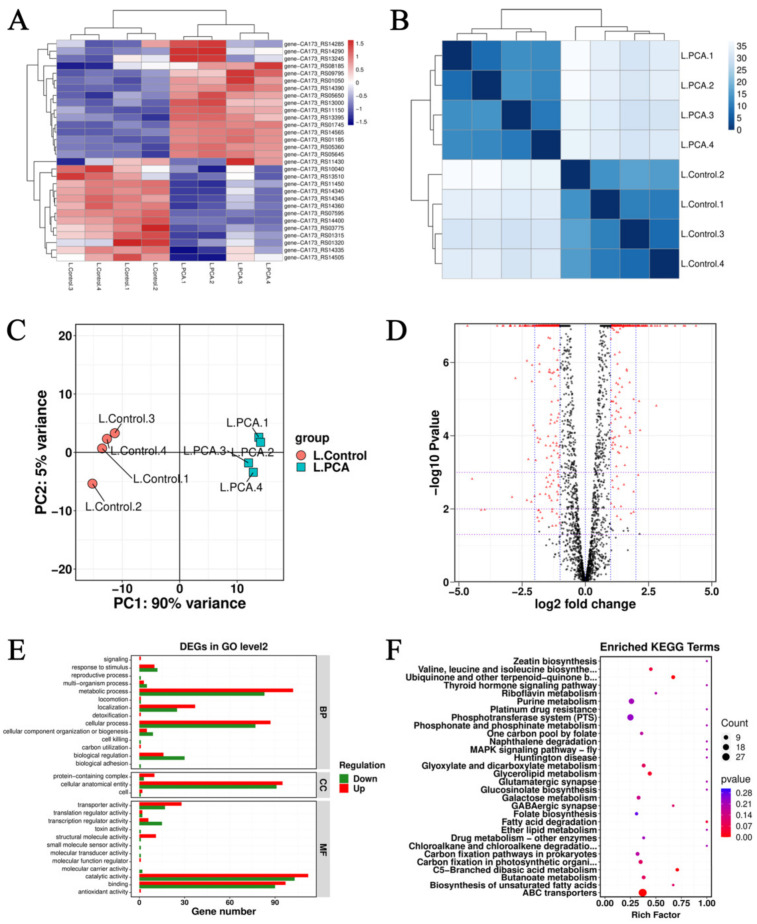
(**A**) Cluster diagram of gene expression levels of *L. monocytogenes* ATCC 19114 under the action of PCA. (**B**) Gene distance heat map of *L. monocytogenes* ATCC 19114 with PCA. (**C**) PCA analysis of the transcriptome of the control and the experimental group of *L. monocytogenes* ATCC 19114. (**D**) Volcano plot of DEGs in *L. monocytogenes* ATCC 19114 between the control and treatment. (**E**) Bar plot of GO enrichment results. (**F**) Statistical enrichment of differential expression genes in KEGG pathways.

Hierarchical clustering analysis was used to determine the relationship of genome-wide expression profiles between control and experimental samples (Figure 3A). In the experimental samples represented by blue and red, the redder the color, the higher the gene expression, and the bluer the color, the lower the gene expression. It was found that the four biological replicates in the control and experimental groups were situated close to each other, and there was a similar pattern between the differentially expressed genes (DEGs); however, there were significant differences between the DEGs in the control and experimental groups. Figure 3B shows a hierarchical clustering analysis of the relationships of all samples using the expression of all genes. Where the color blocks represent the distance values, the darker the color indicates the closer the distance between samples and the higher the similarity, and the lighter the color, the more distant the distance. The results indicate a good correlation between samples and high similarity of gene expression patterns for subsequent gene annotation analysis.

The transcriptome data from the control and experimental groups were used for principal component analysis (Figure 3C). The results showed similar PC scores between the four biological replicate samples in each group, indicating that the data for each sample were reliable in the group. However, the data of *L. monocytogenes* in the treated and control groups were separated to a large extent, indicating a significant effect on *L. monocytogenes* after treatment with PCA.

We used changes in |log_2_FC| ≥ 1 and FDR ≤ 0.05 as the threshold values to define the DEGs in the experimental versus the control group. The overall distribution is shown in Figure 3D (each dot in the figure represents a gene, red dots represent DEGs after the screening, and black dots represent genes that did not meet the screening criteria, where the left side is a down-regulated expression and the right side is an up-regulated expression). Among the 562 genes were differentially expressed significantly (*p* < 0.5) in samples of *L. monocytogenes* after 4 h of PCA treatment, of which 249 DEGs were up-regulated and 313 DEGs were down-regulated. The degree of differential expression was relatively concentrated for most genes, but the differential expression ploidy of a proportion of genes showed a high degree of dispersion, and the highly dispersed expression may have been significantly disturbed by PCA.

#### 3.7.2. GO and KEGG Analysis of DEGs

DEGs were annotated and analyzed using the GO database, and the distribution of DEGs in the transcriptome data of PCA-treated *L. monocytogenes* at GO level 2 is shown in Figure 3E. DEGs were annotated to a total of 29 functional groups, of which 14 cellular processes, 3 biological processes, and 12 molecular functions were changed. We found that most DEGs were associated with response to stimulus, metabolic process, localization, cellular process, and biological regulation for the biological process, mostly exhibiting gene up-regulation properties. Among the cellular components, the cellular anatomical entity was the most abundant. In the molecular function, DEGs were mainly distributed in transporter activity, transcription regulator activity, structural molecule activity, catalytic activity, and binding, mostly showing gene up-regulation. The changes in these genes suggest that PCA treatment of *L. monocytogenes* leads to a cellular defense response, possibly through intracellular metabolic regulation to improve resistance to adverse external environments.

The results of KEGG annotation were classified based on the bioinformatics database comparing DEGs obtained by transcriptome sequencing. Figure 3F shows that DEGs were significantly involved in metabolic pathways such as ABC transporters, glycerolipid metabolism, phosphotransferase system (PTS), and purine metabolism. In addition, according to the analysis of the data shown in Table 2, the DEGs that were up-regulated by exposure to PCA were mainly situated in the amino acid biosynthesis, glycerolipid metabolism, purine metabolism, ABC transporters, ribosome, and aminoacyl-tRNA biosynthesis pathways. The down-regulated DEGs were mainly associated with carbohydrate metabolism, pyrimidine metabolism, PTS, two-component system, and quorum sensing. These findings demonstrate that PCA can affect genes related to metabolic pathways in *L. monocytogenes*, thereby inhibiting the growth of *L. monocytogenes*.

#### 3.7.3. DEGs Associated with Amino Acid Metabolism

The release of intracellular substances from PCA-treated *L. monocytogenes* resulted in a series of amino acid metabolism disorders. Bacteria are highly adaptable, complex systems that can change their overall metabolic capacity for optimum growth in response to changing environmental conditions [36]. As shown in Table 2, under PCA stimulation, *L. monocytogenes* regulates several pathways related to amino acid synthesis and metabolism. DEGs for alanine, aspartate, and glutamate metabolism (CA173_RS11265, *argH*, *purF*, CA173_RS13115), as well as *trpA*, CA173_RS14770, and *trpB* for regulating glycine, serine, and threonine metabolic signals, were overexpressed, indicating that PCA-treated *L. monocytogenes* cells appeared to have abnormal amino acids in an unbalanced state. In the presence of disturbed amino acid metabolism, *L. monocytogenes* treated with PCA activate several amino acid biosynthetic pathways to maintain cell survival [37]. Analysis of pathways related to amino acid metabolism showed that DEGs involved in valine, leucine, and isoleucine biosynthesis (*leuD*, *ilvB*, *ilvC*, *leuC*, *leuB*), and *trpA*, CA173_RS08780, *trpB* for phenylalanine, tyrosine, and tryptophan biosynthesis, and CA173_RS11265 and *argH* for arginine biosynthesis were significantly up-regulated, while CA173_RS07790 involved in lysine biosynthesis was significantly down-regulated. This result suggests that the synthesis and metabolic pathways of various amino acids are disturbed, and *L. monocytogenes* may modify the structure and function of cell membranes by up-regulating amino acid metabolism in response to the damage caused by PCA.

#### 3.7.4. DEGs Associated with Nucleotide Metabolism

Nucleotide metabolism plays a significant role in the production of purine and pyrimidine molecules for DNA replication, RNA synthesis, cell proliferation, differentiation, and apoptosis [38,39]. As shown in Table 2, DEGs of several important systems associated with DNA replication and repair were significantly altered under PCA stress, including mainly 14 purine metabolism, 6 pyrimidine metabolism, 5 aminyl-tRNA biosynthesis, 2 protein export, and 1 base excision repair-related DEGs. In most bacteria, synthesis or access to purines and pyrimidines, which are the basis of nucleotides, is required for survival [40]. Cells in a normal state have purine and pyrimidine metabolism in balance with in vivo metabolic regulation, and metabolic imbalance caused by an abnormality at one point will result in abnormal nucleic acid metabolism [41]. In PCA, DEGs under PCA treatment were significantly enriched in purine and pyrimidine metabolic pathways, implying that PCA may ultimately inhibit cell growth by disrupting the DNA replication and repair system of *L. monocytogenes*.

As a crucial location for processing genetic information, ribosomes directly regulate cellular protein synthesis and indirectly regulate life processes. Disrupting or interfering with ribosomal components would significantly alter ribosomes’ ability to perform as intended [42]. We found that several genes associated with ribosomes were significantly up-regulated after PCA treatment (Table 2). This indicates that the normal protein synthesis process of *L. monocytogenes* was disturbed, and ribosome function was hampered. In addition, the aminoacyl-tRNA biosynthetic pathway is a component of the translational pathway of protein synthesis [43]. Under PCA treatment, multiple DEGs associated with the aminoacyl-tRNA biosynthetic pathway in *L. monocytogenes* were all up-regulated in transcription. Disruption of the amyl-tRNA synthesis pathway affects related protein synthesis pathways, which in turn affects cell proliferation and signal transduction [44]. Also, the expression of genes linked to protein export (folding, sorting, and degradation) and base excision repair (DNA replication and repair) (*sipY*, *yidC*, CA173_RS08830) was disrupted. The disturbance of genetic information processing may be mainly attributed to the disruption of ribosomal components caused by PCA. These results suggest that DNA replication and repair systems are impaired by exposure to PCA, which inhibits protein biosynthesis and metabolism, and ultimately inhibits *L. monocytogenes* growth.

#### 3.7.5. DEGs Associated with Membrane Transport

During the inhibition of *L. monocytogenes* by PCA, most DEGs are associated with the PTS and ABC transporter proteins. The PTS is a bacterial transport system primarily in charge of absorbing significant amounts of carbohydrates during energy transport and catalyzing their conversion to the corresponding phosphates [45]. It has been shown that PTS is essential for biofilm formation, regulation and coordination of carbon and nitrogen metabolism, sugar transport, and several processes related to metabolic and transcriptional regulation [46]. As shown in Table 2, the DEGs associated with the PTS system were mostly down-regulated, indicating that PCA suppressed carbon catabolism in *L. monocytogenes* and could not maintain cell energy and nutrients required for normal survival, thus inhibiting the growth of *L. monocytogenes*.

Except for PTS, ABC transporter proteins were significantly differentially expressed at transcript levels after PCA treatment (Table 2). ABC transporter proteins are a class of transporter proteins with nucleotide-binding domains (NBDs) in charge of ATP binding and hydrolysis that carry out an array of tasks in cells [47]. Transporter proteins can couple the energy produced by ATP hydrolysis and transport a sea of molecules by reversing the concentration gradient, importing essential nutrients, and exporting toxic molecules to the cell [48,49]. In bacteria, essential metals are often used to facilitate biological processes, and iron is incorporated into numerous biological processes due to its oxidation state and rich environmental levels, and iron scarcity disrupts ATP, nucleotide synthesis, and enzyme function [50]. In our study, the migration of some metal cations changed significantly, including CA173_RS10595 (iron ABC transporter permease), CA173_RS00780 (metal ABC transporter permease), CA173_RS10590 (iron ABC transporter permease), CA173_RS11765 (iron ABC transporter permease), CA173_RS10045 (metal ABC transporter ATP-binding protein), and CA173_RS08995 (metal ABC transporter substrate-binding protein). Bacteria are remarkably adaptive to changes in environment and stress, and their survival and proliferation depend on intracellular metal concentrations, whose homeostasis is regulated by transport systems through uptake and removal of metals, and have evolved many resistance mechanisms [51,52]. In addition, amino acids are a source of carbon and nitrogen necessary for bacterial metabolic mutual interactions. Three amino acid-related means of transport were also significantly altered, including CA173_RS05445 (glycine betaine/L-proline ABC transporter ATP-binding protein), CA173_RS05455 (glycine/betaine ABC transporter substrate-binding protein), CA173_RS05450 (proline/glycine betaine ABC transporter permease), CA173_RS12580 (amino acid ABC transporter ATP-binding protein), CA173_RS13040 (methionine ABC transporter ATP-binding protein). The increased uptake of glycine and proline, and the decreased uptake of methionine suggest that PCA may mediate carbon and nitrogen metabolism in *L. monocytogenes* by altering glycine, proline, and methionine.

Inorganic phosphate (Pi) is a substance that plays a crucial role in the metabolism of cellular energy and is found in phospholipids, nucleic acids, and other cellular components. The phosphate-specific transport (Pst) system gene cluster *PstSCAB* is a key gene in the Pi uptake system [53] among the Pst transport proteins. The transmembrane structural domain (TMD) consists of *PstA* and *PstC*, while *PstB* is the dimeric nucleotide-binding structural domain that drives the transport. *PstS* is the peripheral phosphate-binding protein that supplies Pi to TMD [54]. In the transcriptome analysis results, the upregulation of genes associated with the Pst system (*PstA*, *PstB*, and *PstC*) suggests that PCA causes a decrease in energy metabolism in *L. monocytogenes*, providing additional energy to sustain the cell through the Pst system. In addition, ABC transporter proteins function as efflux pumps, and bacteria can actively expel toxic foreign substances from the cells [55]. The significant upregulation of ABC transporter protein expression after PCA treatment is a self-protective mechanism to better adapt to the environment, which to some extent alleviates the damage and other toxic effects caused by PCA on *L. monocytogenes*. This also indicates that PCA has caused damage to cell membranes.

#### 3.7.6. DEGs Associated with Two-Component Systems

In bacteria, the two-component system is the primary signaling switch that controls signaling events, and it includes signaling pathways associated with drug resistance, oxidative stress, pathogenicity, and biofilm formation [56]. In PCA-treated *L. monocytogenes*, the expression of genes associated with the two-component system was altered. The two-component system senses and responds to changes in the cell’s external environment, which in turn leads to changes in gene expression regulation and physiological behavior of the cell. Among them, flagellar motility can facilitate the bacterial acquisition of essential nutrients from the surrounding environment. As shown in Table 2, we identified up-regulated genes *fliI*, CA173_RS03900 associated with flagellar assembly and function that mediate motility of *L. monocytogenes*. *FliI* protein is an ATPase that provides energy for flagellin output, the CA173_RS03900 gene is a member of the *FliH*/*SctL* family protein, and the *FliH* protein is a negative regulator of *FliI*. Thus, the up-regulation of *FliI* indicates the increased output of *L. monocytogenes* flagellar-associated proteins, and *L. monocytogenes* obtain more nutrients from the surrounding environment by migrating to more locations. These changes in DEGs associated with flagellar assembly suggest that PCA interferes with the flagellar assembly process, resulting in reduced motility of *L. monocytogenes*.

In addition, chemotaxis is the directed movement of *L. monocytogenes* sensing material stimuli towards a more favorable environment. Therefore, adequate expression of flagellar genes and proper assembly of flagellar proteins facilitate the movement and exert the chemotactic behavior of *L. monocytogenes*. The bacterial chemotaxis system is a typical coupling protein-dependent signaling system that is essential for bacterial adherence and colonization [57]. The relevant gene involved encodes a methyl-acceptor chemotaxis protein (CA173_RS03940), and the down-regulation of this gene indicated that PCA reduced the adhesion, motility, and chemotactic ability of *L. monocytogenes*. These results suggest that the chemotaxis and motility of *L. monocytogenes* were inhibited by PCA treatment, thereby suppressing their growth.

The two-component system also includes genes associated with population sensing. Population sensing is an intercellular communication process that is generally used by bacteria to control the formation of biofilms in response to variations in cell density and species composition in microbial communities [58]. The biofilm is considered a bacterial community attached to the bacterial surface and enclosed in a self-produced biopolymer matrix that offers the bacteria a haven [59]. Upon treatment with PCA, most of the DEGs associated with biofilm formation were down-regulated, resulting in disruption of biofilm protection, and this disruption inhibited the growth of *L. monocytogenes* until death.

#### 3.7.7. DEGs in Other Key Metabolic Pathways

During the inhibition of *L. monocytogenes* by PCA, the energy metabolism of *L. monocytogenes* was somewhat hindered, and the intracellular energy metabolism was disturbed. However, by enhancing some carbohydrate metabolism, amino acid metabolism, lipid metabolism, and other substances to supplement the energy supply to the cells to maintain the energy necessary for the survival of *L. monocytogenes* and the synthesis of secondary metabolites. This indicates that the cells adjust their physiological functions to cope with the adverse changes in the surrounding environment. Meanwhile, the signaling pathways involved in *L. monocytogenes* were also inhibited by PCA. When cells are stimulated by PCA, they are involved in regulating and controlling biological processes such as cell proliferation, migration, and apoptosis through the interaction of various signaling pathways. In addition, DEGs associated with the cell wall, cell membrane, and their internal components were also significantly expressed, suggesting that PCA inhibits *L. monocytogenes* by disrupting the integrity and structure of the cell wall and cell membrane, thereby inhibiting growth.

### 3.8. Simulation Study of PCA on the Growth Inhibition of L. monocytogenes in the Cooked Chicken Breast Meat

*L. monocytogenes* is a representative pathogenic bacterium in food, which easily contaminates meat, dairy products, and aquatic products. In this experiment, we constructed a model for the growth of *L. monocytogenes* in cooked chicken breast after PCA treatment. As shown in Table 1, the bacterial population increased by 0.57 log_10_ CFU/g in the untreated group, 0.3 log_10_ CFU/g in the MIC group, and 0.2 log_10_ CFU/g in the 2 × MIC group treated with PCA during the 5-day storage period. From these results, it can be seen that PCA effectively inhibited *L. monocytogenes* in cooked chicken breast meat. For the experimental data in Table 1, a quadratic model of the logarithm of *L. monocytogenes* colonies log_10_ (N/(CFU/g)) on the independent variables time (A), temperature (B) and PCA concentration (C) were developed using the experimental optimization software Design Expert 11:

*Y* = 5.38 + 0.1813 *A* + 0.0775 *B* − 0.3538 *C* + 0.015 *AB* − 0.0825 *AC* − 0.035 *BC* − 0.0328 *A^2^* − 0.0403 *B^2^* + 0.1373 *C^2^*, *R*^2^ = 0.9788 (1)

**Figure 4:**
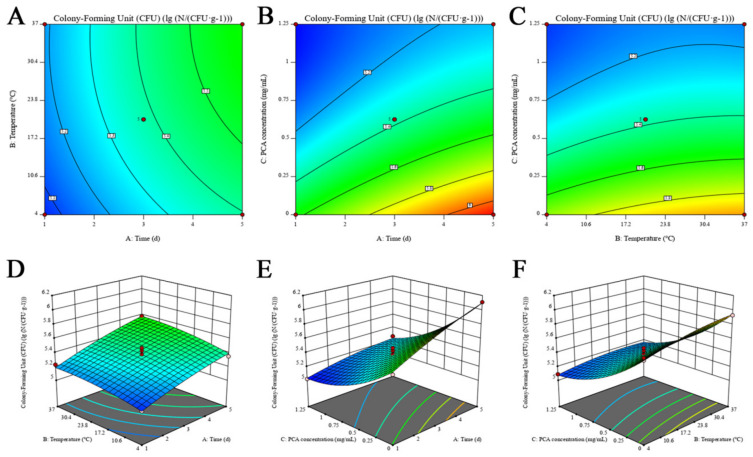
Contour plots and response surface plots illustrate the effect arising from the interaction between PCA and variables on the growth of *L. monocytogenes* ATCC 19114 in cooked chicken breast meat. The contour plots illustrate the effect of interactions between (**A**) time and temperature, (**B**) time and PCA concentration, and (**C**) temperature and PCA concentration on *L. monocytogenes* growth in cooked chicken breast meat. The response surface plots illustrate the effect of interactions between (**D**) time and storage temperature, (**E**) time and PCA concentration, (**F**) temperature and PCA concentration on *L. monocytogenes* growth in the cooked chicken breast meat.

Table 3 presents the MANOVA of the quadratic regression model. As indicated by the results of this table, the model was confirmed to be significant (*p* < 0.01) and could effectively reflect the experimental results. To be specific, the correlation coefficient *R*^2^ = 0.9788 and the correction coefficient *R*^2^_Adj_ = 0.9516 suggested that the measured values of *L. monocytogenes* fit well with the predicted values of *L. monocytogenes*, and the changing pattern of different conditions on the logarithm of *L. monocytogenes* colonies in the cooked chicken breast meat could be evaluated more accurately. Moreover, *p* < 0.01 for A and C factors and *p* < 0.05 for B factor revealed that the time, the temperature, and the PCA concentration significantly affected the total number of *L. monocytogenes* colonies in the chicken breast meat. *p* < 0.05 for the interaction term AC reflected a significant interaction between the time and the PCA concentration. The above findings can be illustrated by the response surface, as presented in Figure 4. And the results of the present model simulation are similar to the model of cranberry pomace extract on the growth of *L. monocytogenes* in cooked ham and pork burgers studied by Tamkutė et al. [60]. Furthermore, the present model may provide theoretical support for the use of PCA as a preservative to extend the shelf life of cooked chicken breasts.

### 3.9. Effect of PCA on the Color of the Cooked Chicken Breast Meat

**Figure 5:**
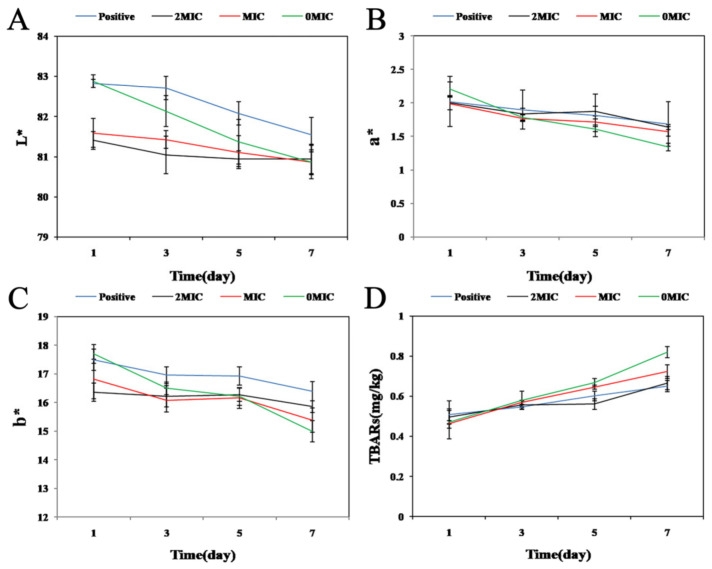
The effects of different concentrations of PCA in the cooked chicken breast meat (**A**) L* value, (**B**) a* value, (**C**) b* value, and (**D**) TBARS content.

Muscle color is one of the important evaluation indicators for judging muscle senses [61]. The color change of the cooked chicken breast meat in the storage process is closely related to the storage time [24]. Currently, the most frequent method to measure the color of the cooked chicken breast is by colorimeter [61]. From Figure 5A–C, the L*, a*, and b* values of the cooked chicken breast meat decreased with increasing refrigeration time, but the L* values in the PCA-treated group were more stable than those in the control group (Figure 5A). As shown in Figure 5B, the a* values of the cooked chicken breast meat in the control group decreased more significantly than those in the test group, and the cooked chicken breast meat was dark yellow and gave off a foul odor in the late storage period. Also, In Figure 5C, the b* values of MIC and the control groups decreased sharply at 5–7 days, while the b* values of 2 × MIC and positive groups changed less. Thus, the effect of PCA on the color stabilization of the cooked chicken breast meat throughout the storage process was 2 × MIC > MIC > 0 × MIC indicating that PCA could better maintain the color change of the cooked chicken breast meat and prolong its shelf life. In addition, Wang et al. observed that L* and a* values decreased and b* values increased during storage of chicken breast slices at 4 °C, while the addition of cinnamon essential oil nanoemulsion (CON) effectively stabilized the color change of chicken breast [62]. The changes in the color characteristics of chicken breast meat may be caused by lipid oxidation or by the color of the natural plant extracts themselves and the amount added [63,64].

### 3.10. Effects of PCA on TBARS in the Cooked Chicken Breast Meat

Lipid oxidation of meat and meat products can result in undesirable changes in color, taste, and texture, which cause spoilage and deterioration of meat products while reducing their nutritional value. MAD refers to the degradation products of lipid peroxide and peroxide formed by the oxidation of polyunsaturated fatty acids [65]. In particular, TBARS are adopted to reflect lipid oxidation as a measure of food quality. Figure 5D presents the changes in TBARS values for the cooked chicken breast meat with PCA addition, where the TBARS values for the cooked chicken breast meat increased with increasing storage days. The TBARS value for the control group was 0.47 mg MDA/kg and increased to 0.82 mg MDA/kg by 7 d. The TBARS value of MIC increased from 0.46 mg MDA/kg to 0.72 mg MDA/kg. The TBARS value of 2 × MIC was elevated from 0.49 mg MDA/kg to 0.66 mg MDA/kg. Moreover, the control group increased faster than MIC and 2 × MIC. It was therefore proven that PCA could be effective in slowing down the degree of fat oxidation in cooked chicken breast meat and avoiding the decomposition of sourness and unacceptable odor to maintain the good flavor of chicken breasts in the storage process. Furthermore, Al-Hijazeen et al. effectively reduced the TBARS values of chicken breast and thigh meat during refrigeration at 4 °C with the use of Oregano EO and tannic acid. Cooked meat was reported to be more susceptible to lipid oxidation due to the denaturation of antioxidant enzymes and structural damage of membranes [26].

### 3.11. Effects of PCA on TPA in the Cooked Chicken Breast Meat

TPA refers to using a texture analyzer to simulate human oral chewing behavior and measure the texture characteristics of food based on hardness, adhesiveness, springiness, cohesiveness, chewiness, and resilience. TPA serves as one of the essential indicators to evaluate meat products [25,66]. Table 4 lists the changes in TPA parameters of the cooked chicken breast meat by adding different concentrations of PCA during refrigeration. It can be seen from the table that the hardness and chewiness of the cooked chicken breast meat showed an overall increasing trend with the increase in storage time. However, springiness, cohesiveness, and resilience did not change significantly. The change in hardness tended to increase and then decrease, with the control group showing the most significant increase from 4013.82 ± 96.83 to 6585.17 ± 225.27. Moreover, chewiness indexes also increased significantly during storage (*p* < 0.05), with the PCA-treated group showing a more stable change in hardness and masticatory properties than the untreated group. The above results revealed that PCA could stably maintain the changes in hardness and chewiness of the cooked chicken breast meat in the storage period. Moreover, the samples had increased hardness and chewiness, which could arise from the denaturation or aggregation of proteins. The meat products underwent shrinkage changes in some structures of the meat components by the heat treatment, thus leading to the hardness of the meat pieces [67,68].

### 3.12. Effects of PCA on Sensory Characteristics in the Cooked Chicken Breast Meat

**Figure 6:**
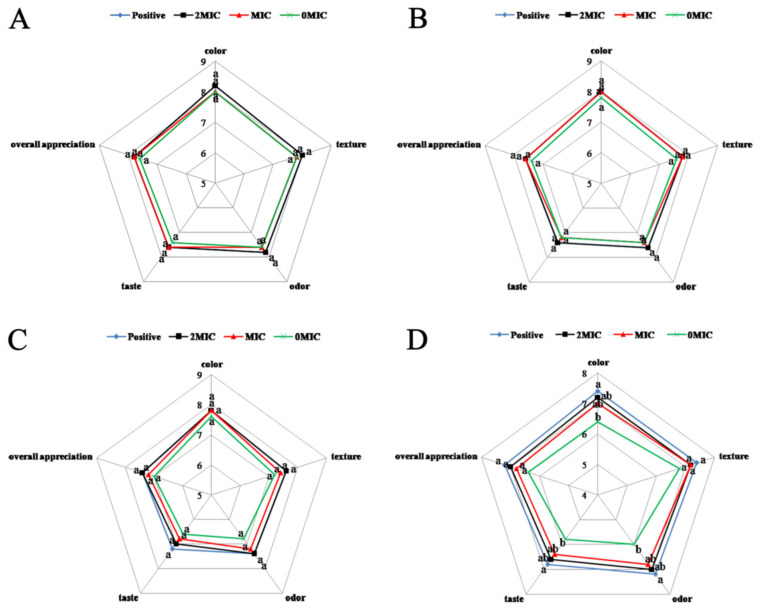
Radar plot of sensory properties of PCA applied to the cooked chicken breast meat at different storage periods. (**A**) 1st, (**B**) 3rd, (**C**) 5th, and (**D**) 7th day. ^a,b^ Values with different letters under the same characteristic represent significant differences (*p* < 0.05).

The results of the sensory evaluation of chicken breast patties (Figure 6) showed that no significant effects on color, texture, odor, taste, and overall appreciation of the PCA-treated chicken breast patties occurred throughout the storage period (*p* > 0.05). However, the color, odor, and taste of the chicken breast patties were rated higher in the experimental group than in the control group on 7 d, and the chicken breast patties in the non-PCA treated group had emitted a foul odor, which was consistent with the results of the color and texture measurements of the chicken breasts described above. These results indicate that PCA improves the sensory characteristics of chicken breast patties and can be applied to chicken breast meat to be accepted by consumers.

## 4. Conclusions

These results indicate that PCA exhibited effective antibacterial activity against *L. monocytogenes*. Firstly, PCA caused cell membrane hyperpolarization decreased intracellular ATP concentration, and lowered pH_in_ by disrupting the integrity of the *L. monocytogenes* cell membrane, and CLSM and FEG-SEM identified the damage to the cell membrane. Secondly, transcriptome results showed that PCA interferes with various amino acid synthesis and metabolic pathways in *L. monocytogenes* cells, impairs DNA replication and repair systems, inhibits carbon catabolism, causes damage to cell membranes, and fails to maintain the energy and nutrients required for normal cell survival, thereby inhibiting the growth of *L. monocytogenes*. Ultimately, the addition of PCA significantly inhibited the growth of *L. monocytogenes* and the formation of MDA, a fat oxidation product, during the storage of cooked chicken breast and improved the color and texture of the cooked chicken breast meat. Accordingly, PCA, a natural antioxidant with significant antibacterial activity and antioxidant capacity, which can effectively extend the shelf life of cooked chicken breast meat and improve the safety and quality of food.

## Figures and Tables

**Table 1:** Box–Behnken experimental design and responses.

Trials	*A* (Day)	*B* (°C)	*C* (mg/mL)	log_10_ (N/(CFU/g))
1	1	4	0.625	5.06
2	5	4	0.625	5.35
3	1	37	0.625	5.23
4	5	37	0.625	5.58
5	1	20.5	0	5.54
6	5	20.5	0	6.11
7	1	20.5	1.25	5.02
8	5	20.5	1.25	5.26
9	3	4	0	5.75
10	3	37	0	5.93
11	3	4	1.25	5.09
12	3	37	1.25	5.13
13	3	20.5	0.625	5.29
14	3	20.5	0.625	5.32
15	3	20.5	0.625	5.38
16	3	20.5	0.625	5.43
17	3	20.5	0.625	5.47

**Table 2:** Details of DEGs annotated by KEGG.

Gene	ko_Annotation	Log_2_ FC	*p*-Value	Description
CA173_RS11265	Alanine, aspartate, and glutamate metabolism	1.081957	1.18 × 10^−7^	argininosuccinate synthase
*argH*	Alanine, aspartate, and glutamate metabolism	1.061599	2.35 × 10^−12^	argininosuccinate lyase
*purF*	Alanine, aspartate, and glutamate metabolism	1.019488	6.40 × 10^−17^	amidophosphoribosyltransferase
CA173_RS13115	Alanine, aspartate, and glutamate metabolism	1.097684	4.90 × 10^−17^	glutamate decarboxylase
CA173_RS14770	Glycine, serine, and threonine metabolism	1.275227	1.94 × 10^−15^	glycerate kinase
*trpA*	Glycine, serine, and threonine metabolism	1.184578	3.52 × 10^−5^	tryptophan synthase subunit alpha
*trpB*	Glycine, serine, and threonine metabolism	1.191056	8.71 × 10^−6^	tryptophan synthase subunit beta
*leuB*	Valine, leucine, and isoleucine biosynthesis	1.971215	2.36 × 10^−5^	3-isopropylmalate dehydrogenase
*leuC*	Valine, leucine, and isoleucine biosynthesis	1.206237	1.43 × 10^−11^	3-isopropylmalate dehydratase large subunit
*leuD*	Valine, leucine, and isoleucine biosynthesis	1.905842	5.80 × 10^−13^	3-isopropylmalate dehydratase small subunit
*ilvB*	Valine, leucine, and isoleucine biosynthesis	1.172642	0.000109	biosynthetic-type acetolactate synthase large subunit
*ilvC*	Valine, leucine, and isoleucine biosynthesis	1.858133	4.95 × 10^−13^	ketol-acid reductoisomerase
CA173_RS08780	Phenylalanine, tyrosine, and tryptophan biosynthesis	1.401025	1.79 × 10^−5^	phosphoribosylanthranilate isomerase
CA173_RS07790	Lysine biosynthesis	-1.21078	2.38 × 10^−8^	aspartate-semialdehyde dehydrogenase
*rpmC*	Ribosome	1.016667	1.16 × 10^−5^	50S ribosomal protein L29
*rpmI*	Ribosome	1.297633	1.50 × 10^−7^	50S ribosomal protein L35
*rplB*	Ribosome	1.016719	5.72 × 10^−6^	50S ribosomal protein L2
*rplO*	Ribosome	1.079427	1.03 × 10^−21^	50S ribosomal protein L15
*rplQ*	Ribosome	1.083072	1.17 × 10^−19^	50S ribosomal protein L17
*rplX*	Ribosome	1.072298	9.74 × 10^−11^	50S ribosomal protein L24
*rpsJ*	Ribosome	1.017788	1.12 × 10^−7^	30S ribosomal protein S10
*rpsN*	Ribosome	1.75559	7.29 × 10^−5^	30S ribosomal protein S14
*rpsP*	Ribosome	1.414987	9.13 × 10^−17^	30S ribosomal protein S16
*rpsQ*	Ribosome	1.181848	1.02 × 10^−7^	30S ribosomal protein S17
CA173_RS14110	Ribosome	1.295859	3.94 × 10^−6^	type Z 30S ribosomal protein S14
*pheS*	Aminoacyl-tRNA biosynthesis	1.742778	7.10 × 10^−46^	phenylalanine--tRNA ligase subunit alpha
*pheT*	Aminoacyl-tRNA biosynthesis	1.571565	6.94 × 10^−40^	phenylalanine--tRNA ligase subunit beta
*glyQ*	Aminoacyl-tRNA biosynthesis	1.176408	6.23 × 10^−16^	glycine--tRNA ligase subunit alpha
*glyS*	Aminoacyl-tRNA biosynthesis	1.309933	1.64 × 10^−18^	glycine--tRNA ligase subunit beta
*thrS*	Aminoacyl-tRNA biosynthesis	1.117751	8.79 × 10^−17^	threonine--tRNA ligase
*sipY*	Protein export	−1.48206	9.88 × 10^−17^	type I signal peptidase SipY
*yidC*	Protein export	−1.01195	4.81 × 10^−14^	membrane protein insertaseYidC
CA173_RS08830	Base excision repair	−2.94502	6.17 × 10^−50^	DNA-3-methyladenine glycosylase I
CA173_RS02040	Phosphotransferase system (PTS)	−1.12959	0.013481	PTS fructose transporter subunit IIB
CA173_RS10815	PTS	−1.60707	6.74 × 10^−16^	PTS sugar transporter subunit IIB
CA173_RS11305	PTS	−3.27105	2.50 × 10^−11^	PTS sugar transporter subunit IIA
CA173_RS02610	PTS	−1.13536	2.40 × 10^−14^	PTS galactitol transporter subunit IIC
CA173_RS01540	PTS	−2.96986	7.42 × 10^−8^	PTS lactose/cellobiose transporter subunit IIA
CA173_RS01825	PTS	−1.31607	0.011838	PTS sugar transporter subunit IIA
CA173_RS11300	PTS	−2.76471	2.69 × 10^−6^	PTS sugar transporter subunit IIB
CA173_RS14630	PTS	−1.9508	2.59 × 10^−23^	PTS sugar transporter subunit IIC
CA173_RS02605	PTS	−1.79285	0.003955	PTS sugar transporter subunit IIB
CA173_RS04710	PTS	−1.8375	0.021573	PTS sugar transporter subunit IIB
CA173_RS14910	PTS	−1.30713	0.010148	PTS sugar transporter subunit IIB
CA173_RS02585	PTS	−1.41589	1.57 × 10^−6^	PTS sugar transporter subunit IIA
CA173_RS11295	PTS	−2.5824	1.23 × 10^−42^	PTS galactitol transporter subunit IIC
CA173_RS00135	PTS	−1.22299	1.57 × 10^−16^	beta-glucoside-specific PTS transporter subunit IIABC
CA173_RS00170	PTS	1.335248	0.000102	PTS sugar transporter subunit IIC
CA173_RS15010	PTS	1.22531	3.64 × 10^−6^	PTS lactose/cellobiose transporter subunit IIA
CA173_RS15095	PTS	1.323207	5.42 × 10^−12^	PTS sugar transporter subunit IIA
CA173_RS12525	PTS	2.010962	1.56 × 10^−34^	fructose-specific PTS transporter subunit EIIC
CA173_RS15020	PTS	1.404133	0.000196	PTS sugar transporter subunit IIB
*PstA*	ABC transporters	2.070414	2.26 × 10^−12^	phosphate ABC transporter permeasePstA
*PstB*	ABC transporters	1.519848	3.54 × 10^−13^	phosphate ABC transporter ATP-binding protein PstB
*PstC*	ABC transporters	1.375445	1.20 × 10^−7^	phosphate ABC transporter permease subunit PstC
*fliI*	Flagellar assembly	1.501106	2.54 × 10^−9^	flagellar protein export ATPaseFliI
CA173_RS03900	Flagellar assembly	1.149952	3.41 × 10^−13^	FliH/SctL family protein
CA173_RS03940	Bacterial chemotaxis	−1.35731	4.07 × 10^−29^	methyl-accepting chemotaxis protein
CA173_RS13115	Quorum sensing	1.097684	4.90 × 10^−17^	glutamate decarboxylase
CA173_RS00240	Quorum sensing	1.205978	2.72 × 10^−15^	accessory gene regulator ArgB-like protein
CA173_RS00250	Quorum sensing	−1.09844	8.53 × 10^−13^	sensor histidine kinase
CA173_RS12515	Quorum sensing	−1.87055	3.90 × 10^−7^	competence protein ComK
*plcB*	Quorum sensing	−1.00494	3.00 × 10^−8^	phosphatidylcholine phospholipase C
*hly*	Quorum sensing	−1.42193	1.39 × 10^−25^	cholesterol-dependent cytolysin listeriolysin O

**Table 3:** The regression model for predicting the best conditions for *L. monocytogenes* inhibition using the analysis of variance.

Source	Sum of Squares	df	Mean Square	F-Value	*p*-Value
Model	1.43	9	0.1591	35.98	<0.0001
A-A	0.2628	1	0.2628	59.43	0.0001
B-B	0.0481	1	0.0481	10.87	0.0132
C-C	1	1	1	226.39	<0.0001
AB	0.0009	1	0.0009	0.2035	0.6655
AC	0.0272	1	0.0272	6.16	0.0421
BC	0.0049	1	0.0049	1.11	0.3275
A^2^	0.0045	1	0.0045	1.02	0.3459
B^2^	0.0068	1	0.0068	1.54	0.2542
C^2^	0.0793	1	0.0793	17.94	0.0039
Residual	0.031	7	0.0044		
Lack of Fit	0.0087	3	0.0029	0.5192	0.6915
Pure Error	0.0223	4	0.0056		
Cor Total	1.46	16			

**Table 4:** Chicken breast texture test results with PCA added at different storage times.

TPA	Time/d	Positive Control	2 × MIC	MIC	Control
Hardness	1	4837.5 ± 53.09 ^c^	4649.28 ± 195.73 ^c^	4320.58 ± 239.06 ^d^	4013.82 ± 96.83 ^d^
	3	6231.74 ± 236.2 ^b^	7002.53 ± 112.7 ^a^	6678.25 ± 196.37 ^b^	7097.74 ± 244.8 ^b^
	5	7068.13 ± 102.94 ^a^	7574.16 ± 398.98 ^a^	7605.47 ± 32.98 ^a^	7999.56 ± 110.85 ^a^
	7	6514.4 ± 186.08 ^b^	6289.94 ± 219.33 ^b^	6011.5 ± 63.03 ^c^	6585.17 ± 225.27 ^c^
Springiness	1	0.94 ± 0.01	0.97 ± 0.01	0.97 ± 0.01	0.95 ± 0.02
	3	0.94 ± 0.02	0.95 ± 0.01	0.97 ± 0.02	0.98 ± 0.01
	5	0.94 ± 0.02	0.97 ± 0.01	0.96 ± 0.01	0.98 ± 0.01
	7	0.96 ± 0.01	0.97 ± 0.01	0.97 ± 0.01	0.97 ± 0.01
Cohesiveness	1	0.75 ± 0.01	0.77 ± 0.01	0.8 ± 0.02	0.8 ± 0.05
	3	0.77 ± 0.06	0.75 ± 0.01	0.79 ± 0.1	0.77 ± 0.02
	5	0.76 ± 0.09	0.79 ± 0.06	0.76 ± 0.09	0.76 ± 0.01
	7	0.78 ± 0.02	0.8 ± 0.07	0.76 ± 0.06	0.74 ± 0.01
Chewiness	1	3434.17 ± 52.46 ^c^	3604.26 ± 227.76 ^b^	3452.85 ± 112.93 ^b^	2928.25 ± 116.22 ^c^
	3	4310.81 ± 101.25 ^b^	5037.57 ± 133.96 ^a^	4908.74 ± 294.64 ^a^	4934.46 ± 148.19 ^b^
	5	4492.62 ± 75.85 ^b^	5461.53 ± 321.74 ^a^	5267.5 ± 87.36 ^a^	6614.91 ± 329.4 ^a^
	7	4888.11 ± 235.65 ^a^	5581.4 ± 327.7 ^a^	5016.59 ± 81.48 ^a^	5902.15 ± 451.93 ^a^
Resilience	1	0.36 ± 0.01	0.37 ± 0.04	0.37 ± 0.02	0.37 ± 0.01
	3	0.36 ± 0.03	0.34 ± 0.01	0.36 ± 0.05	0.37 ± 0.04
	5	0.34 ± 0.04	0.34 ± 0.03	0.34 ± 0.05	0.35 ± 0.02
	7	0.36 ± 0.02	0.37 ± 0.03	0.33 ± 0.03	0.32 ± 0.02

The positive control was ampicillin added at a concentration of 0.1 mg/mL. Each value is expressed as mean ± standard deviation (*n* = 3). ^a–d^ Values in the same column with different letters represent significant differences for the same factor at different storage times (*p* < 0.05).

## Data Availability

Data is contained within the article.
